# Blood or Urine IP-10 Cannot Discriminate between Active Tuberculosis and Respiratory Diseases Different from Tuberculosis in Children

**DOI:** 10.1155/2015/589471

**Published:** 2015-08-05

**Authors:** Linda Petrone, Angela Cannas, Francesco Aloi, Martin Nsubuga, Joseph Sserumkuma, Ritah Angella Nazziwa, Levan Jugheli, Tedson Lukindo, Enrico Girardi, Klaus Reither, Delia Goletti

**Affiliations:** ^1^Translational Research Unit, Department of Epidemiology and Preclinical Research, National Institute for Infectious Diseases (INMI), 00149 Rome, Italy; ^2^Italian Association for Solidarity among People (AISPO), P.O. Box 7146, Kampala, Uganda; ^3^St. Francis Nsambya Hospital, P.O. Box 7146, Kampala, Uganda; ^4^Swiss Tropical and Public Health Institute, Medical Services and Diagnostic Department, 4002 Basel, Switzerland; ^5^University of Basel, 4003 Basel, Switzerland; ^6^Ifakara Health Institute, Bagamoyo Research and Training Center, P.O. Box 78373, Bagamoyo, Tanzania; ^7^Department of Epidemiology and Preclinical Research, National Institute for Infectious Diseases (INMI), 00149 Rome, Italy

## Abstract

*Objectives*. Interferon-*γ* inducible protein 10 (IP-10), either in blood or in urine, has been proposed as a tuberculosis (TB) biomarker for adults. This study aims to evaluate the potential of IP-10 diagnostics in children from Uganda, a high TB-endemic country. *Methods*. IP-10 was measured in the blood and urine concomitantly taken from children who were prospectively enrolled with suspected active TB, with or without HIV infection. Clinical/microbiological parameters and commercially available TB-immune assays (tuberculin skin test (TST) and QuantiFERON TB-Gold In-Tube (QFT-IT)) were concomitantly evaluated. *Results*. One hundred twenty-eight children were prospectively enrolled. The analysis was performed on 111 children: 80 (72%) of them were HIV-uninfected and 31 (27.9%) were HIV-infected. Thirty-three healthy adult donors (HAD) were included as controls. The data showed that IP-10 is detectable in the urine and blood of children with active TB, independent of HIV status and age. However, although IP-10 levels were higher in active TB children compared to HAD, the accuracy of identifying “active TB” was low and similar to the TST and QFT-IT. *Conclusion*. IP-10 levels are higher in children with respiratory illness compared to controls, independent of “TB status” suggesting that the evaluation of this parameter can be used as an inflammatory marker more than a TB test.

## 1. Introduction

Childhood tuberculosis (TB) is an urgent problem to address, especially in high endemic countries. In 2013, about 550,000 children globally were estimated to have had active TB, resulting in 80,000 deaths [1]. TB diagnosis in children is difficult [2], as clinical symptoms are nonspecific, samples are difficult to obtain, and disease is often paucibacillary [3, 4]. The tuberculin skin test (TST) is recommended for latent TB infection (LTBI) diagnosis in children younger than 5 years of age [5]. Recently, interferon-*γ* release assays (IGRAs), such as QuantiFERON TB-Gold In-Tube (QFT-IT), have been recommended for diagnosing LTBI in children older than 5 years of age in western countries [5, 6].

The interferon- (IFN-) *γ* inducible protein 10 (IP-10) is a chemokine expressed by antigen-presenting cells in response to IFN-*γ* that attracts activated T-cells to foci of inflammation [7]. IP-10 has been shown to be involved in the response to TB, as shown by the presence of IP-10-positive cells in the bronchoalveolar lavage [8] or lymph node aspirate specimens with granulomas in patients with active TB [9]. Moreover, IP-10 was found to be increased in the unstimulated day-1 plasma of children with active TB compared to subjects without active TB [10–12] and similarly in TB adults [13]. IP-10 is also considered to be an alternative marker to IFN-*γ* in the assays based on the QFT-IT format [14–16].

Besides blood, IP-10 can be detected in the urine of patients with active TB and has been shown to decrease after efficacious therapy [17]. Urine biomarkers may offer several advantages over blood since urine is easier to collect, especially in children. Moreover, biorisks (risk associated with biological materials and/or infectious agents) are lower in urine, and no special equipment or specialized healthcare personnel are required for collection. All these factors are relevant, especially in resource-poor high endemic TB countries.

To date, there is no published evidence of utilizing IP-10 as a biomarker for TB concomitantly evaluated in urine and blood. Therefore, in this prospective study, urine and blood IP-10 were evaluated in children from a high TB-endemic country enrolled with suspected active TB, with or without Human Immunodeficiency Virus (HIV) infection. The results were analyzed in relationship to clinical/microbiological parameters and also with commercially available TB-immune assays since the IP-10-based test is an immunological assay.

## 2. Methods

### 2.1. Study Population

This prospective study was carried out in children from 1 month to 16 years of age who attended the St. Francis Nsambya Hospital, Kampala, Uganda, from May 10, 2011, until September 4, 2012, with signs or symptoms that suggested TB. Children were followed up for a minimum of 5 months. At least one of the following eligibility criteria had to be met: persistent, nonremitting cough for more than 14 days that did not respond to antibiotics; repeated episodes of fever within the previous 14 days that did not respond to antibiotics, after malaria had been excluded; weight loss or failure to thrive during the previous 3 months; and signs and symptoms that suggested extrapulmonary tuberculosis. Subjects who had received TB treatment in the previous 12 months were excluded; therefore none of the enrolled subjects were undergoing TB therapy at the time of recruitment. The children were referred from peripheral health facilities and local hospitals. Written informed consent was obtained from adults and from a literate parent or legal guardian of the children.

Since healthy children were not enrolled for ethical issues, healthy adult donors (HAD) were included as controls. The Uganda National Council for Science and Technology approved the study protocol.

### 2.2. Classification and Reference Standard

Classification of children with or without active TB was based on the criteria shown in the list below. Children with respiratory infections other than TB were classified as “respiratory diseases” and included “pneumonia” (*n* = 24), “respiratory tract infections” (*n* = 20), and “*miscellanea*” (*n* = 17) (Table 1).

*Tuberculosis Categories*

*(A) Culture-Confirmed Tuberculosis*. Symptoms suggestive of tuberculosis plus liquid or solid culture positive for* M. tuberculosis*.

*(B) Highly Probable Tuberculosis.* Symptoms suggestive of tuberculosis plus one of the following:

- chest radiograph strongly indicating active tuberculosis and confirmed by 2 independent reviewers;
- histology/cytology showing typical morphology;
- fluorescent or acid-fast bacilli on microscopy.

*(C) Probable Tuberculosis.* Cervical lymph node mass (greater than 2*∗*2 cm) with resolution on TB therapy

- or abdominal mass or ascites with abdominal lymphadenopathy or ultrasound scan and resolution on TB therapy;
- or clinical picture of meningitis associated with CSF changes and/or CT scan findings consistent with TB (after other likely causes have been excluded);
- or suggestive symptoms, but CXR “uncertain” for active tuberculosis or discordance between independent reviewers, no alternative diagnosis established and complete symptomatic or radiographic resolution on TB treatment.

*(D) Not Tuberculosis (Controls).* Alternative diagnosis established, TB workup negative and clinically well after 3–6 months follow-up without TB treatment.

*(E) Indeterminate*. Any other possible combination of results and/or loss to follow-up after recruitment.

### 2.3. Clinical and Laboratory Procedures

Clinical procedures at enrolment comprised demographics, medical history, physical examination, HIV testing [ARCHITECT HIV Ag/Ab Combo (Abbott Diagnostics, Wiesbaden, Germany)], CD4 T-cell count, QFT-IT (Qiagen, Hilden, Germany), TST, and chest radiographs. Chest radiographs were classified as strongly indicative, uncertain, or nonactive TB by two independent experts from whom all clinical and diagnostic information was masked. The TST, performed only in children, consisted of an intradermal injection of 10 TU Purified Protein Derivative (Statens Serum Institute, Copenhagen, Denmark). The TST was defined positive at ≥10 mm induration in HIV-uninfected children or ≥5 mm in HIV-infected children [18]. If feasible, at least three induced or two expectorated respiratory specimens were obtained on consecutive days. Fine-needle aspiration biopsies of enlarged lymph nodes were done when TB lymphadenopathy (LAD) was suspected [19]. Clinical samples (sputum, lymph node aspirate, and blood) were assessed by microscopy after Ziehl-Neelsen staining. At least one sample was inoculated on both liquid (BACTEC MGIT 960 Mycobacterial Detection System, Becton Dickinson, Franklin Lakes, NJ, USA) and solid media (Lowenstein-Jensen culture). All tests, including the IP-10 assay, were done by trained laboratory technical staff blinded to clinical information and radiological results. Results from established diagnostic procedures were made available to support clinical management in accordance with national and international guidelines; however, the results of the experimental IP-10 assay were not released.

### 2.4. Sample Collection and IP-10 Measurement

IP-10 was measured in blood and urine samples concomitantly collected as described [20, 21]. As spot morning urine samples were available, urine IP-10 levels were normalized and reported as a ratio to creatinuria [21].

### 2.5. Statistical Analysis

Analysis was carried out with SPSS v.19 for Windows (SPSS Italia SRL, Bologna, Italy) and Prism 6 software (Graphpad Software 6.0, San Diego, USA). Medians and interquartile ranges (IQR) were calculated for continuous measures, and the Chi-square was used for dichotomous measures. The Kruskall-Wallis test and Mann-Whitney *U* test were used for comparisons among several groups or pairwise comparisons, respectively. *p* values ≤0.05 or resulting from Bonferroni correction were considered significant. The cut-off value was defined by a receiver operating characteristics (ROC) analysis. Spearman rank correlation was used to correlate continuous variables; *r*_*s*_ > 0.7 was considered a high correlation, 0.7 < *r*_*s*_ > 0.5 a moderate correlation, and *r*_*s*_ < 0.5 a low correlation.

## 3. Results

### 3.1. Population Characteristics

**Figure 1:**
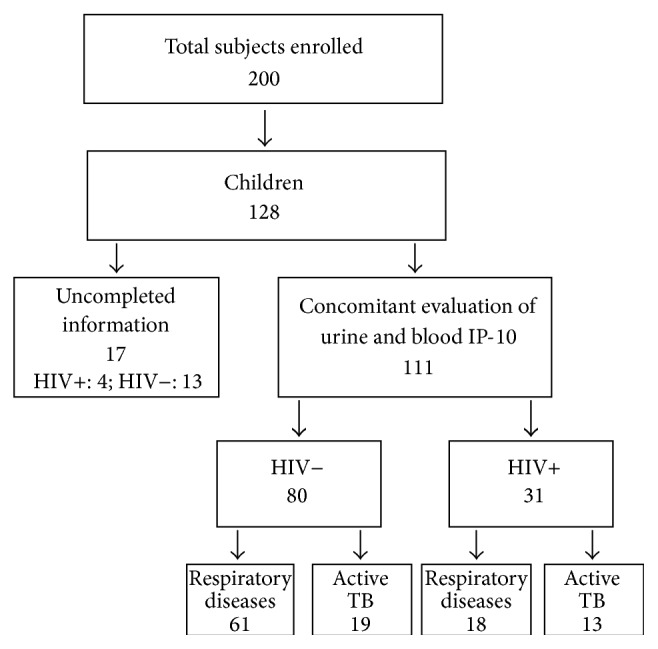
Flow chart of the study participants evaluated.

We prospectively enrolled 128 children (Figure 1). Seventeen (13.3%) were excluded from the study because the children left the hospital before a definitive diagnosis was reached or for “nonconclusive findings” (“indeterminate”). Therefore, the analysis was performed on 111 (86.7%) children for whom a concomitant evaluation of blood and urine IP-10 was available. Among them, 80 (72.1%) were HIV-uninfected and 31 (27.9%) were HIV-infected. We also included 33 HAD HIV-uninfected subjects, as the healthy group control. This approach was based on the fact that children and adults with active TB showed similar IP-10 levels in both urine and blood compartments (unpublished data).

The HIV-uninfected children defined as having “active TB” (*n* = 19) had a median age of 8 years (IQR: 2–14) and the urine parameters evaluated were in the normal range (Table 1). “Active TB” children were further classified into “culture-confirmed TB” (*n* = 7), “highly probable TB” (*n* = 7), and “probable TB” (*n* = 5). The HIV-uninfected children in the “respiratory diseases” group (*n* = 61) had a median age of 4 years (IQR: 2–8) and were mainly males [34 (56.7%)] (Table 1). The HIV-infected children defined as having “active TB” (*n* = 13) had a median age of 9 years (IQR: 4–14) and the urinary parameters evaluated were in the normal range (Table 1). “Active TB” children were classified as having “highly probable TB” (*n* = 10) and “probable TB” (*n* = 3). The HIV-infected children in the “respiratory diseases” group (*n* = 18) had a median age of 5 years (IQR: 3–8) and their clinical characteristics were similar to those recorded in the HIV-uninfected children with respiratory diseases (Table 1). Similar to the HIV-uninfected children, the “respiratory diseases” children were classified as having “pneumonia” (*n* = 8), “respiratory tract infections” (*n* = 4), and “*miscellanea”* (*n* = 6) (Table 1).

The HAD had a median age of 26 years (IQR: 23–30) and the urinary parameters evaluated were in the normal range (Table 1).

### 3.2. Blood and Urine IP-10 Levels Were Significantly Increased in Children with “Active TB” compared to Healthy Subjects, but They Did Not Distinguish between “Active TB” and Other Respiratory Diseases

**Figure 2:**
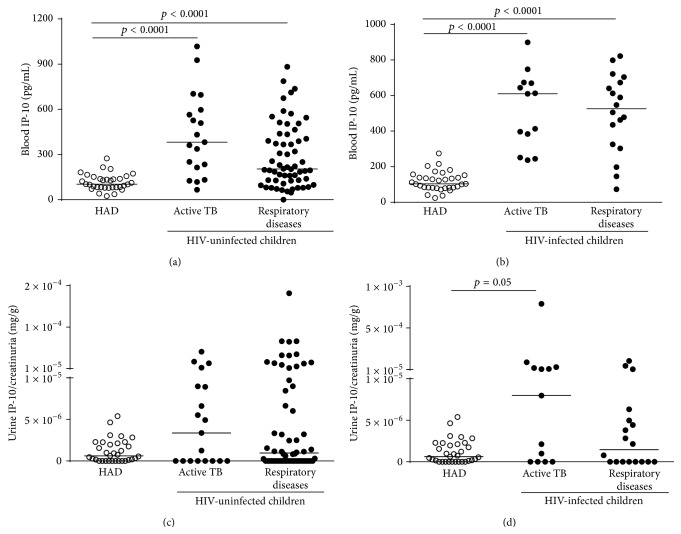
Blood and urine IP-10 levels are significantly increased in children with “active TB” compared to healthy adult donors (HAD). (a-b) Blood IP-10 levels were significantly increased in the children with a diagnosis of “active TB” and “respiratory diseases” compared to HAD, independent of HIV status. (c-d) Urine IP-10 levels were increased in the children diagnosed with “active TB” compared to the other two groups (HAD and “respiratory diseases”). IP-10 ELISA was performed in plasma and urine, and urine IP-10 was normalized with the creatinuria levels. The horizontal lines represent the median; statistical analysis was performed using the Mann-Whitney test with Bonferroni correction and *p* value was considered significant if <0.016. IP-10: IFN-*γ* inducible protein 10; HAD: healthy adult donors; TB: tuberculosis; HIV: Human Immunodeficiency Virus.

Blood IP-10 levels were significantly increased in “active TB” children and “respiratory diseases” children compared to HAD, independent of HIV status (HIV-uninfected “active TB” children versus HAD: *p* ≤ 0.0001; HIV-uninfected “respiratory diseases” children versus HAD: *p* ≤ 0.0001; HIV-infected “active TB” children versus HAD: *p* ≤ 0.0001; HIV-infected “respiratory diseases” children versus HAD: *p* ≤ 0.0001) (Figures 2(a) and 2(b); Table 2). Importantly, no differences between the “active TB” and “respiratory diseases” groups were found.

Before analyzing the IP-10 results in urine, to ascertain that the kidney functions were similar among the different groups, we evaluated the correlation between urine IP-10 and several urinary parameters (specific gravity, proteinuria, leucocytes, nitrates, and pH). We only found a significant but low correlation between urine IP-10 and specific gravity (*p* ≤ 0.0001, *r*_*s*_ = 0.39). No significant differences among the urinary parameters were found among the different groups evaluated (data not shown).

Similar to the blood results, the urine IP-10 levels were increased in “active TB” children compared to the other two groups, independent of HIV status (Figures 2(c) and 2(d), Table 2) although the differences were not significant.

### 3.3. Blood or Urine IP-10 Levels Are Not Correlated with Age in Children

**Figure 3:**
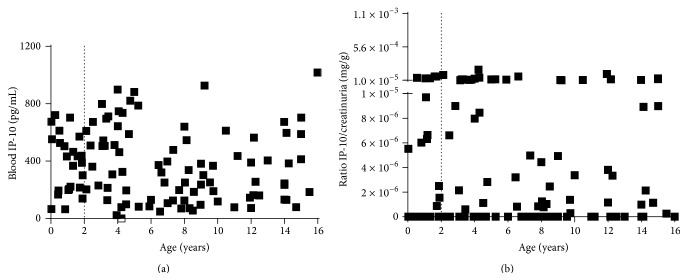
Blood and urine IP-10 do not correlate with age in children. (a) Spearman rank correlation (*r*_*s*_) of blood IP-10 and age of children (*p* = 0.1, *r*_*s*_ = −0.1). (b) Spearman rank correlation (*r*_*s*_) of urine IP-10 and age in children (*p* = 0.8, *r*_*s*_ = −0.01). ELISA was performed in blood or urine and urine IP-10 was normalized with the creatinuria levels. Statistical analysis was performed using the Spearman rank correlation test and *p* value was considered significant if <0.05. IP-10: IFN-*γ* inducible protein 10.

We then evaluated if there was a correlation between blood and urine IP-10 levels and if IP-10 measurements in blood or urine depended on age. A significant but low correlation between blood and urine IP-10 levels (*p* = 0.004, *r*_*s*_ = 0.27) (data not shown) was found. Importantly, no correlation between the levels of IP-10 and age in either compartments (blood IP-10: *p* = 0.1, *r*_*s*_ = −0.1; urine IP-10: *p* = 0.8, *r*_*s*_ = −0.01) was found. Therefore both blood and urine IP-10 can be detected in children younger than 2 years of age (Figures 3(a) and 3(b)).

### 3.4. QFT-IT and TST Commercial Tests in Children and Adults

We then evaluated the performance of commercial immune tests for LTBI diagnosis, using active TB as a surrogate marker for the sensitivity of the test. In the HIV-uninfected children with active TB, the QFT-IT was positive in 9 (47.4%) patients, negative in 7 (36.8%), and indeterminate in 1 (5.3%); the TST was positive in 11 (57.9%) (Table 3). In the HIV-uninfected children with “respiratory diseases,” the QFT-IT was positive in 6 (10.2%), negative in 50 (84.7%), and indeterminate in 3 (5.1%); the TST was positive in 10 (16.7%) (Table 3).

In the HIV-infected children with active TB, the QFT-IT was positive in only 2 out of 12 (16.7%) and negative in 10 (83.3%). Similarly, the TST was positive in only 3 (23.1%) (Table 3). None of the HIV-infected children with “respiratory diseases” were positive to QFT-IT, 14 (82.4%) subjects were QFT-IT-negative, and 3 (17.6%) were scored as indeterminate; the TST was positive in only 1 (5.6%) (Table 3).

Regarding the HAD among the 32/33 tested, 13 (40.6%) were QFT-IT positive (Table 3).

### 3.5. Insufficient Accuracy of the IP-10-Based Test and Commercial Tests QFT-IT and TST for Active TB Diagnosis

Finally, we evaluated the potential use of IP-10 measurement in blood and urine for active TB diagnosis. Based on the differences found between “active TB” children and HAD, we performed a ROC analysis to evaluate the diagnostic performance of the blood IP-10 test. Significant results in the AUC analysis were found (AUC, 0.88; 95% confidence interval (CI), 0.77–0.99, *p* ≤ 0.0001). A cut-off value was chosen to maximize the sum of sensitivity and specificity. The cut-off point of 209.1 pg/mL predicted “active TB” with 79.0% sensitivity (95% CI, 54.4–94%) and 93.9% specificity (95% CI, 79.8–99.3%). Thus, we evaluated if this cut-off point discriminated “active TB” from “respiratory diseases” in the HIV-uninfected and HIV-infected children. The sensitivity and the specificity of blood IP-10 were 79% and 53%, respectively, in the HIV-uninfected children, and 100% and 17%, respectively, in the HIV-infected children (Table 4).

A similar approach was used to evaluate the accuracy of urine IP-10. No significant AUC results were found (AUC, 0.61; 95% CI, 0.42–0.81, *p* = 0.2). A cut-off point of 3.2*e* − 006 mg/g predicted “active TB” with 52.6% sensitivity (95% CI, 28.9–75.6%) and 93.9% specificity (95% CI, 79.8–99.3%). However, the performance of the urine IP-10-based test to identify “active TB” was inferior, as the sensitivity and the specificity of urine IP-10 were 53% and 66%, respectively, in the HIV-uninfected children, and 54% and 61%, respectively, in the HIV-infected children (Table 4).

Evaluation of IP-10 either in blood or in urine is an immune-based test. Therefore, we compared the accuracy of these experimental tests with the accuracy of the commercial immune-based test for TB. The sensitivity of the QFT-IT and TST for active TB diagnosis was comparable in both the HIV-uninfected (53% and 61%, resp.) and HIV-infected children (17% and 23%, resp.). QFT-IT specificity for active TB diagnosis was also similar in the HIV-uninfected and HIV-infected children (83% and 82%, resp.) and increased when the indeterminate results were excluded from the analysis (Table 4). TST specificity for active TB diagnosis was 82% in the HIV-uninfected children and 94% in the HIV-infected children.

## 4. Discussion

In this prospective study performed in a high TB-endemic country, we demonstrated that IP-10 is detectable in the blood and urine of children with active TB, independent of age. IP-10 levels were significantly different in active TB children compared to healthy donors. However, the accuracy of this immune experimental test to identify “active TB” was low and similar to other commercial immune-based tests for TB such as the TST and QFT-IT. Blood and urine IP-10 levels were found higher in diseased people compared to controls, suggesting that the evaluation of this parameter can be used as an inflammatory marker to assess the state of immune activation. Indeed, IP-10 is involved in leucocyte activation and is inducible by IFN-*γ* production, in the context of a long-lasting immune response. It is remarkable that IP-10 can be detected in urine, independent of a child's age. Therefore, it may be possible to propose IP-10 as a potential inflammatory marker for clinical workup, taking into account that urine IP-10 can be assessed easier than the established blood tests such as C-Reactive Protein and Erythrocyte Sedimentation Rate.

The measurement of IP-10 in blood has been recommended for monitoring disease activity and efficacy of TB therapy in adults [13], as IP-10 levels were found increased in patients with active TB at baseline and declined after successful treatment [13]. IP-10 has been proposed as a TB biomarker, also for children. Indeed, it is increased in unstimulated day-1 plasma in children with active disease compared to subjects without active TB [10–12]. However, like IFN*γ*, IP-10 does not distinguish between active TB and LTBI [12]. Here we confirm these results in a larger cohort of patients using a biological specimen that is easier to obtain,* ex vivo* unstimulated plasma, in both the HIV-infected and HIV-uninfected subjects. Moreover, IP-10 was shown, for the first time to our knowledge, to be detectable in the urine of children with active TB, to be uncorrelated with urinary parameters (proteinuria, leucocytes, nitrates, and pH), with the exception of specific gravity, as shown in adults [17], and to significantly correlate with blood IP-10, as demonstrated in adults with Hepatitis C Virus (HCV) infection [21]. Urine IP-10 levels were found to be increased in the “active TB” group compared to the healthy adult donors, independent of HIV status. This difference reached significance only in the HIV-infected children. This is probably due to the fact that the level of inflammation in the HIV-infected subjects with “active TB” status is higher than in the “HIV-uninfected” ones, as evidenced in the literature [22, 23].

IP-10 was also found increased in children with diseases other than active TB. In this study, the “respiratory diseases” group included mainly children with pneumonia or respiratory tract infections. This is likely due to the fact that IP-10 is a chemokine mediating leukocyte trafficking and cell activation, crucial during inflammation [24], and therefore detected in the patients with lung infections/diseases enrolled within the “respiratory diseases” group of the present study. Thus, our data confirm that IP-10 cannot be considered a specific marker for mycobacterial infection or disease and can be measured during both bacterial and viral infections, as previously demonstrated for HIV [25] or HCV infection [21, 26].

To evaluate the accuracy of the experimental IP-10-based immune assay in diagnosing active TB, we compared the results with those obtained by the TST and QFT-IT, two commercial immune-based tests for TB. All assays showed poor sensitivity and specificity for active TB, especially in the HIV-infected children. Recent meta-analyses show that the overall sensitivity of the QFT-IT or TST for diagnosing active TB in children varies between 50 and 100% [27]. All these data together indicate that better assays are needed for TB diagnosis in children.

In our study, urine IP-10 was not detected in some patients. Different factors may explain these data. In particular, blood IP-10 concentration can affect urine IP-10 detection, as IP-10 is a small protein and, once filtered in the glomerulus, can easily be reabsorbed by renal tubules; therefore, if low IP-10 levels are detected in blood, even lower IP-10 levels will be measured in urine. Moreover, IP-10 was evaluated in a spot urine sample in this study. Concentrating spot urine samples or evaluating the morning urine samples may possibly overcome this issue.

The main drawback of this study is that, due to ethical reasons, healthy children were not included in our study and therefore, the HAD were used as surrogate controls. However, we think this approach was reasonable, as we found similar IP-10 results in both the urine and blood compartments of children and adults with active TB (unpublished data). Furthermore, other immune-based tests (e.g., QFT-IT) are employed in adults and children using the same cut-off point which was generated in a cohort of healthy adults. Moreover, the small sample size within the “active TB” subgroups hampered us from drawing any definitive intergroup conclusion. However, the design of the prospective study that aimed to evaluate IP-10 as biomarker in subjects with suspected TB, the unique approach of the concomitant evaluation of the experimental immune diagnostic tests for TB in blood and urine, the comparison with commercial tests for TB diagnosis, and the systematic analysis give strength to this work and substantiate the findings.

## 5. Conclusion

Our data demonstrated that IP-10 is detectable in the blood and urine of children with active TB. However, similar to other immune-based commercial tests such as the TST and QFT-IT, the accuracy of identifying “active TB” is low especially in immune-compromised patients [15, 28–31]. This is the first study which has taken a holistic approach to scouting for biomarkers by concomitantly evaluating the urine and blood in children, with and without HIV infection, from a high TB-endemic country. These results, although unsatisfactory for TB diagnosis, contribute to better understanding of the potential of evaluating blood and urine IP-10 as an inflammatory marker.

## Figures and Tables

**Table 1:** Demographic and clinical characteristics of the subjects enrolled excluding those lost to follow-up.

	HIV-uninfected children					HIV-infected children					Healthy adult donors
	Culture-confirmed	Highly probable	Probable	Total active TB	Respiratory diseases	Culture-confirmed	Highly probable	Probable	Total active TB	Respiratory diseases	
*n* (%)	7	7	5	19	61	0	10	3	13	18	33

Median age (IQR)	12 (9–15)	8 (1–14)	3 (1–8)	8 (2–14)	4 (2–8)	—	8 (1–14)	14	9 (4–14)	5 (3–8)	26 (23–30)

Male gender (%)	3 (42.8)	4 (57.1)	2 (40.0)	9 (47.4)	34 (56.7)	—			4 (30.8)	8 (44.4)	12 (46.2)

TB localization											
Pulmonary (PTB)	7 (100)	6 (85.7)	5 (100)	18 (94.7)	—	0 (0)	10 (100)	3 (100)	13 (100)	—	—
Extrapulmonary (ETB)	0 (0)	0 (0)	0 (0)	0 (0)	—	0 (0)	0 (0)	0 (0)	0 (0)	—	—
PTB and EPT	0 (0)	1 (14.3)	0 (0)	1 (5.3)	—	0 (0)	0 (0)	0 (0)	0 (0)	—	—

Diseases other than TB											
Pneumonia	—	—	—	—	24 (39.3)	—	—	—	—	8 (44.5)	
Respiratory tract infections	—	—	—	—	20 (32.8)	—	—	—	—	4 (22.2)	
Miscellanea	—	—	—	—	17 (27.9)	—	—	—	—	6 (33.3)	

CD4^+^T-cell/mm^3^ Median (IQR)	—	—	—	—	—	—	388 (328–536)	184 (NA)	386 (243–580)	583 (480–831)	—

Urinary parameters Median (IQR)											
Creatinuria	43.4 (27.0–65.1)	41.1 (26.6–81.6)	18.6 (11.2–36.7)	38.3 (18.6–65.1)	28.7 (12.5–49.6)	—	30.5 (9.4–72.9)	59.8 (NA)	37.7 (13.3–69.6)	37.8 (6.9–48.9)	105.0 (81.4–137.2)
Specific gravity	1.01 (1.01–1.02)	1.02 (1.01–1.02)	1.02 (1.01–1.03)	1.02 (1.01–1.02)	1.01 (1.01–1.02)	—	1.01 (1.01–1.02)	1.02 (NA)	1.01 (1.01–1.02)	1.02 (1.02–1.03)	1.02 (1.02–1.03)
pH	6.0 (6.0–6.5)	6.3 (6.0–7.3)	6.0 (6.0–7.0)	6 (6.0–6.6)	6.5 (6.0–8.0)	—	6.0 (6.0–6.6)	6.0 (NA)	6.0 (6.0–6.8)	6.0 (6.0–6.6)	7.0 (6.0–8.0)

TB: tuberculosis; IQR: interquartile range; HIV: Human Immunodeficiency Virus; QFT-IT: QuantiFERON TB-Gold In-Tube; TST: tuberculin skin test.

**Table 2:** IP-10 values in blood and urine of children with or without active TB stratified by HIV infection. Healthy adult donors were included as controls.

	HIV-uninfected children						HIV-infected children					Healthy adult donors
	Culture-confirmed	Highly probable	Probable	Total active TB	Respiratory diseases		Culture-confirmed	Highly probable	Probable	Total active TB	Respiratory diseases	
*n*	7	7	5	19	61		—	10	3	13	18	33

Blood IP-10 Median (IQR)	563.9 (381.9–926.6)	432.2 (234.4–596.8)	213.9 (95.9–349.2)	381.9 (213.9–596.8)	204.1 (126.4–426.6)		—	611.2 (249.7–688.9)	396.9 (383.6–673.6)	609.8 (317.6–671.5)	525.9 (319.7–680.6)	103.3 (81.9–145.3)

Urine IP-10 Median (IQR)	4.9*e* − 006 (0–1.2*e* − 005)	3.4*e* − 006 (0–2.2*e* − 005)	0 (0–6.1*e* − 006)	3.4*e* − 006 (0–8.9*e* − 006)	9.6*e* − 007 (0–9.5*e* − 006)		—	1.1*e* − 005 (0–5.3*e* − 005)	2.1*e* − 006 (0–1.6*e* − 005)	7.9*e* − 006 (0–3.3*e* − 005)	1.5*e* − 006 (0–5.3*e* − 006)	6.2*e* − 007 (0–2.2*e* − 006)

TB: tuberculosis; IQR: interquartile range; HIV: Human Immunodeficiency Virus.

**Table 3:** QFT and TST results of patients excluding those lost to follow-up.

	HIV-uninfected children						HIV-infected children						Healthy adult donors
	Active TB categories			Total active TB	Respiratory diseases	*p* value	Active TB categories			Total active TB	Respiratory diseases	*p* value	
	Culture-confirmed	Highly probable	Probable				Culture-confirmed	Highly probable	Probable				
*n*	7	7	5	19	60		0	10	3	13	18		33

QFT-IT (%)						**0.001**						0.11	
Performed	5 (71.4)	7 (100.0)	5 (100.0)	17 (89.5)	59 (98.3)		—	9 (90.0)	3 (100.0)	12 (92.3)	17 (94.4)		32 (97.0)
Positive	3 (60.0)	4 (57.1)	2 (40.0)	9 (47.4)	6 (10.2)		—	1 (11.1)	1 (33.3)	2 (16.7)	0 (0)		13 (40.6)
Negative	2 (40.0)	2 (28.6)	3 (60.0)	7 (36.8)	50 (84.7)		—	8 (88.9)	2 (66.7)	10 (83.3)	14 (82.4)		19 (59.4)
Indeterminate	—	1 (14.3)	—	1 (5.3)	3 (5.1)		—	—	—	—	3 (17.6)		—
Not performed	2 (28.6)	—	—	2 (10.5)	1 (1.7)		—	1 (10.0)	—	1 (7.7)	1 (5.6)		1 (3.0)

TST test (%)						**<0.0001**						0.15	
Performed	6 (85.7)	7 (100.0)	5 (100.0)	18 (94.7)	60 (100.0)		—	10 (100.0)	3 (100.0)	13 (100.0)	18 (100)		0
Positive	3 (50.0)	6 (85.7)	2 (40.0)	11 (57.9)	10 (16.7)		—	1 (10.0)	2 (66.7)	3 (23.1)	1 (5.6)		—
Negative	3 (50.0)	1 (14.3)	3 (60.0)	7 (36.8)	50 (83.3)		—	9 (90.0)	1 (33.3)	10 (76.9)	17 (94.4)		—
Not performed	1 (14.3)	—	—	1 (5.3)	—		—	—	—	—	0 (0)		33 (100.0)

TB: tuberculosis; IQR: interquartile range; HIV: Human Immunodeficiency Virus; QFT-IT: QuantiFERON TB-Gold In-Tube; TST: tuberculin skin test.

**Table 4:** Sensitivity and specificity results of IP-10-based tests, QFT-IT, and TST evaluated in children with a diagnosis of “active TB” versus “respiratory diseases.”

	Test	Cut-off	Sensitivity %	Sensitivity % excluding indeterminate results of the QFT	Specificity %	Specificity % excluding indeterminate results of the QFT
HIV status						
HIV-uninfected	IP-10 blood (pg/mL)	209.1	79	NA	53	NA
	IP-10 urine (mg/g)	0.0000032	53	NA	66	NA
	QFT-IT		53	56	83	88
	TST		61	NA	82	NA

HIV-infected	IP-10 blood (pg/mL)	209.1	100	NA	17	NA
	IP-10 urine (mg/g)	0.0000032	54	NA	61	NA
	QFT-IT		17	17	82	100
	TST		23	NA	94	NA

TB: tuberculosis; HIV: Human Immunodeficiency Virus; QFT-IT: QuantiFERON TB-Gold In-Tube; TST: tuberculin skin test; IP-10: interferon-*γ*-inducible protein 10.
